# Disorders of Endogenous and Exogenous Antioxidants in Neurological Diseases

**DOI:** 10.3390/antiox12101811

**Published:** 2023-09-29

**Authors:** Izabela Korczowska-Łącka, Bartosz Słowikowski, Thomas Piekut, Mikołaj Hurła, Natalia Banaszek, Oliwia Szymanowicz, Paweł P. Jagodziński, Wojciech Kozubski, Agnieszka Permoda-Pachuta, Jolanta Dorszewska

**Affiliations:** 1 Laboratory of Neurobiology, Department of Neurology, Poznan University of Medical Sciences, 61-701 Poznan, Polandmikolaj.hurla@gmail.com (M.H.);; 2 Department of Biochemistry and Molecular Biology, Poznan University of Medical Sciences, 61-701 Poznan, Poland; bslowikowski@ump.edu.pl (B.S.); pjagodzi@ump.edu.pl (P.P.J.); 3 Chair and Department of Neurology, Poznan University of Medical Sciences, 61-701 Poznan, Poland; 4 Department of Psychiatry, Psychotherapy and Early Intervention, Medical University of Lublin, 20-059 Lublin, Poland

**Keywords:** oxidative stress, antioxidants, neurological diseases

## Abstract

In diseases of the central nervous system, such as Alzheimer’s disease (AD), Parkinson’s disease (PD), stroke, amyotrophic lateral sclerosis (ALS), Huntington’s disease (HD), and even epilepsy and migraine, oxidative stress load commonly surpasses endogenous antioxidative capacity. While oxidative processes have been robustly implicated in the pathogenesis of these diseases, the significance of particular antioxidants, both endogenous and especially exogenous, in maintaining redox homeostasis requires further research. Among endogenous antioxidants, enzymes such as catalase, superoxide dismutase, and glutathione peroxidase are central to disabling free radicals, thereby preventing oxidative damage to cellular lipids, proteins, and nucleic acids. Whether supplementation with endogenously occurring antioxidant compounds such as melatonin and glutathione carries any benefit, however, remains equivocal. Similarly, while the health benefits of certain exogenous antioxidants, including ascorbic acid (vitamin C), carotenoids, polyphenols, sulforaphanes, and anthocyanins are commonly touted, their clinical efficacy and effectiveness in particular neurological disease contexts need to be more robustly defined. Here, we review the current literature on the cellular mechanisms mitigating oxidative stress and comment on the possible benefit of the most common exogenous antioxidants in diseases such as AD, PD, ALS, HD, stroke, epilepsy, and migraine. We selected common neurological diseases of a basically neurodegenerative nature.

## 1. Introduction

As a result of exposure of organisms to reactive oxidants from internal metabolism and exposure to toxic environmental substances, and nitrogen species, reactive oxygen species (ROS) are generated, causing oxidative stress. On the other hand, controlled production of oxidants serves useful purposes to regulate signaling pathways. An important factor in oxidant signaling and antioxidant defense is reactive cysteine thiol-based redox signaling. The nuclear factor erythroid 2-related factor 2 (Nrf2) is a regulator of cellular resistance to oxidants. Nrf2 controls the expression of antioxidant response-dependent genes to regulate the physiological and pathophysiological outcomes of oxidant exposure [1]. Its role in the antioxidant response is described in detail in the following sections.

Under homeostatic conditions, there is an equilibrium between the formation of free radicals and their removal. Increases in the production of free radicals decreases in the amount of antioxidants, or decreases in the activity of enzymes responsible for free radical removal can result in ineffective elimination of free radicals and ensuing cellular damage. The body’s defense system against oxidative damage can occur in three stages. The first stage involves preventing the formation of free radicals via the enzymatic removal of oxidizing molecules. The second consists of scavengers that interrupt free radical chain reactions. Tertiary prevention involves restoring the correct structure of damaged molecules so that cells do not become more susceptible to future oxidative assault [2].

**Figure 1:**
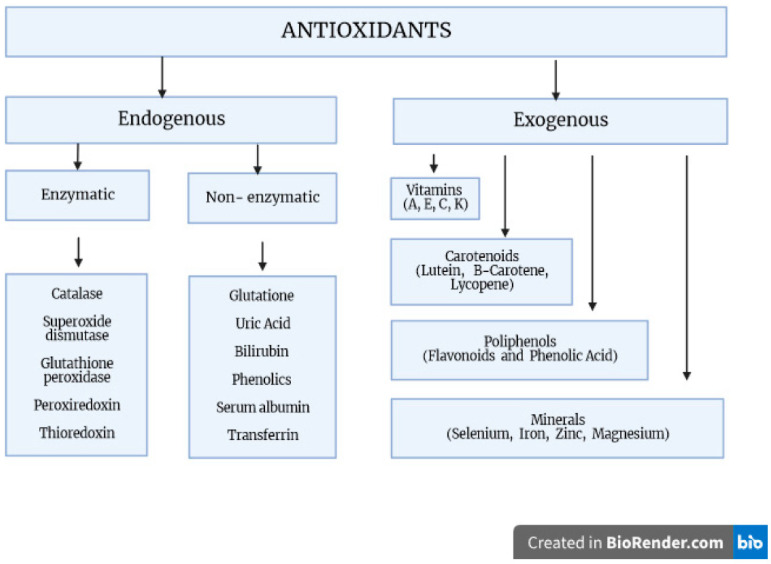
The body’s oxidative stress defense mechanisms. Factors protecting against the generation of free radicals and participating in their transformation into inactive derivatives. These include both exogenous and endogenous compounds, which form a complex antioxidant system with enzymatic and non-enzymatic properties.

Antioxidants are compounds that neutralize reactive chemical species by either directly donating electrons to reduce those species or catalyzing the reactions leading to chemical reduction. Antioxidants can be further broadly classified into two major types: endogenous, i.e., produced by the body, and exogenous, i.e., obtained from diet or supplements. Endogenously occurring enzymes include catalase, superoxide dismutase (SOD), and glutathione peroxidase (GPx). Compounds such as melatonin and glutathione (GSH) are produced endogenously but are also commonly supplemented. Exogenous antioxidants include a wide variety of natural and synthetic compounds such as vitamin C, an acid with three hydroxy moieties present in many fruits and vegetables; carotenoids, compounds that the body converts to the retinoids (vitamin A) required for maintenance of the retinal pigment epithelium in the eye; polyphenols, literally molecules bearing multiple phenol groups thought to be particularly effective in preventing lipid oxidation; sulforaphanes, organosulfur compounds abundant in cruciferous vegetables; and anthocyanins, pigments found in red, blue, and purple fruits and vegetables (Figure 1) [2,3,4].

The current review intends to discuss disturbances of redox homeostasis and antioxidant levels as potential alternative drug strategies for the prevention and effective treatment of common neurological diseases. The level of antioxidants changes as a result of developing neurological diseases. In this article, we present the basic views on oxidative damage to macromolecular compounds and the role of potential antioxidants in reducing neurodegenerative neurological diseases.

## 2. Enzymatic Defenses against Oxidative Stress

### 2.1. Catalase

Catalases may be divided into typical, monofunctional catalases (CATs) or catalase–peroxidases, with the two classes differing based on of the intermediates involved in the catalytic reduction of H_2_O_2_ to H_2_O and O_2_. The said reaction occurs in two steps:

Catalase-Fe (III) + H_2_O_2_ → compound I Compound I + H_2_O_2_ → Catalase-Fe(III) + 2H_2_O + O

CAT consists of four identical, tetrahedrally arranged 60 kDa subunits, each containing a heme group and NADPH in its active center. CATs mainly localize to cellular peroxisomes and, to some extent, the cytosol of mammalian cells but have also been found in other organelles, including mitochondria and chloroplasts. Notably, another form of CATs exists, namely, Mn-CAT, as either iron or manganese can serve as the cofactor in the catalytic breakdown of H_2_O_2_; however, Mn-CAT only exists in bacteria [5,6,7,8,9]. Catalase has the highest turnover rate of all human enzymes, with different reports suggesting that it can degrade between 2.8–40 million molecules of hydrogen peroxide per second [10]. Moreover, unlike glutathione peroxidases, the other enzyme class integral to hydrogen peroxide clearance, which requires GSH to reduce its substrate, catalase functions independently of reducing equivalents, providing robustness to the body’s capacity for eliminating hydrogen peroxide [11,12]. Catalase also uses H_2_O_2_ to oxidize toxins, including phenols, formic acid, formaldehyde, and alcohol.

### 2.2. Superoxide Dismutase

Superoxide dismutase (SOD) is a metalloenzyme that catalyzes the dismutation of superoxide O_2_^•−^ to O_2_ and H_2_O_2_. It exists in several forms that differ in structure and the metal cofactor they contain [13]. In humans, three different forms of SOD have been identified. The copper- and zinc-containing cytosolic SOD (Cu/Zn SOD, SOD1) is a 32 kDa homodimer abundantly expressed in astrocytes and present in plasma, lymph, and cerebrospinal fluids [14]. A minor fraction of SOD1 is found in the mitochondrial intermembrane space [15]. The manganese-containing mitochondrial SOD (MnSOD, SOD2) is an 89 kDa homotetramer [16] also expressed in the central nervous system (CNS) but mainly found in neurons as compared to glia. The third isoform, EC-SOD or SOD3, is an extracellular tetrameric 135 kDa glycoprotein. It has high affinity for heparin and heparin sulfates [17,18]. It is also expressed in the CNS but at lower concentrations than SOD1 and SOD2 [19,20].

### 2.3. Glutathione Peroxidases and Glutathione-S-Transferases

Glutathione peroxidases (GPxs) catalyze the reduction of H_2_O_2_ or organic peroxide (ROOH) to water or alcohol by oxidizing two molecules of GSH:

H_2_O_2_ + 2GSH → GSSG + 2H_2_O ROOH + 2GSH → ROH + 2GSSH + H_2_O

Five out of the eight known human GPx genes encode for selenocysteine-containing proteins. Four of those enzymes exploit selenium in the above catalysis [21]: GPx1, which localizes to the cytosol (75%) and mitochondrial matrix (25%) [22], cytosolic GPx2, extracellular GPx3, and the phospholipid hydroperoxide GPx4 [23]. GPx1, GPx2, and GPx3 are homotetramers, while GPx4 is a monomer. GPx1 activity is expressed in most rodent tissues, with the highest levels occurring in the liver and kidney [24,25,26]. GPx2 is closely related to GPx1 structure and substrate specificity but differs in tissue distribution, being almost exclusively expressed in the gastrointestinal tract [27,28,29]. GPx3 is an extracellular glycoprotein [30,31] that can reduce hydrogen peroxide, lipid hydroperoxides, and phospholipid hydroperoxides, although at lower catalytic rates than other selenoperoxidases [32,33]. It is primarily synthesized in the kidney and exported as a glycoprotein into plasma [34]. GPx4 has cytosolic, mitochondrial, and nuclear isoforms [35]. While only the cytosolic isoform is necessary for embryonic viability, dysfunction of the mitochondrial and nuclear isoforms has been tied with impaired spermatogenesis and neurodegeneration, respectively [21]. Interestingly, GPx4 upregulation has been shown to decrease arachidonic acid oxidation products and NF-kB-mediated inflammatory pathways [36], possibly because it is the only GPx that can reduce complex hydroperoxy lipids [21]. As such, it has also been identified as a key regulator of ferroptosis secondary to Fe-triggered lipid hydroperoxide toxicity [37,38].

Glutathione S-transferases (GSTs) constitute a highly diverse family of enzymes whose primary role lies in detoxifying substrates by conjugating them to GSH, thereby rendering them more soluble and amenable to excretion. Similar to GPxs, GSTs can reduce lipid hydroperoxides, albeit through Se-independent glutathione peroxidase activity [39]. Studies have queried whether certain genetic variants of GST might be risk agents for AD [40] and identified that the GSTM1*0 (homozygous deletion) and GSTP Ile105Val SNP (rs1695) genotypes are associated with AD pathology, with the GSTT1*0 genotype (homozygous deletion) conferring an increased risk for AD in Asian populations only [41,42,43]. Nonetheless, these associations still need to be examined in larger study samples.

### 2.4. Peroxiredoxin

Peroxiredoxins (thioredoxin peroxidases, Prxs) are a family of peroxidases with a conserved cysteine residue integral to the reduction of substrates such as peroxides, alkyl hydroperoxides, and peroxynitrite [44,45,46]. There are three major Prx subclasses based on the number and position of cysteine (Cys) residues involved in catalysis: typical 2-Cys Prxs (Prx1–4), which have conserved N- and C-terminal (CT) Cys residues that are both required for catalytic function [47]; atypical 2-Cys Prxs (Prx5), which utilize a conserved NT Cys and a non-conserved Cys for catalytic activity; and Prx6, which relies solely on one NT Cys for catalysis [48,49,50]. Interestingly, besides supporting catalase and GPxs in forming the body’s arsenal of defenses against oxidative stress, Prxs likely play a role as redox sensors insofar as they are more susceptible to becoming inactivated through oxidation [51]. Indeed, studies in yeast have revealed that hyperoxidized Prx1 triggers a signaling cascade that limits the oxidation of mitochondrial matrix GSH under conditions of high oxidative stress [52,53].

### 2.5. Heme Oxygenase

Heme oxygenase (HO) is an enzyme that catalyzes the rate-limiting step in the degradation of heme to carbon monoxide (CO), biliverdin, and ferrous iron and regulates a wide variety of anti-inflammatory, antioxidant, and antiapoptotic pathways [54,55]. The catalytic reaction implicates cytochrome P450 reductase and reduces molecular oxygen to water at the expense of NADPH oxidation [56]. Two functional isoforms of HO exist. HO-1 is either not transcribed or expressed at very low levels in most tissues under homeostasis conditions but is induced when cells face oxidative stress. The regulation of HO-1 expression is largely determined by the interplay between nuclear factor erythroid 2-related factor 2 (Nrf2), an HO-1 transactivator that exists at low basal levels due to ubiquitination and proteasomal degradation facilitated by Kelch-like ECH-associated protein 1 (KEAP1), and BACH1, a heme sensor that competes with Nrf2 for the HO-1 promoter but serves to inhibit transcription. When intracellular heme levels rise, BACH1 dissociates from the HO-1 promoter, allowing for Nrf2-mediated transactivation to occur. Reactive oxygen species (ROS) also cause conformational changes in KEAP1, ultimately leading to rising concentrations of Nrf2 and, consequently, HO-1. HO-1 is also known to be regulated by hypoxia-inducible factor 1α and anti-inflammatory signaling cascades mediated by AMP-activated protein kinase (AMPK), mitogen-activated protein kinases (MAPKs), and phosphatidylinositol 3-kinase (PI3K). Conversely, HO-2 is expressed in different tissues, including the liver, brain, testes, and vascular system. It causes a sustained generation of the vasodilator CO and, as such, is thought to play a role in maintaining healthy perfusion to the brain [57,58].

The main source of exogenous antioxidants is diet. There are three categories of exogenous, non-enzymatic antioxidants: first are mineral elements such as selenium and zinc; the second is carotenoids, vitamin E and vitamin C; and the third category is phytochemicals/phytonutrients such as flavonols (quercetin, kaempferol and myricetin), isoflavones (genistein, daidzein and glycitein), flavanones (naringenin, eriodictyol and hesperetin), and phenolic acids such as chlorogenic acid, gallic acid and caffeic acid [12].

Genetic variants in genes encoding antioxidant enzymes may increase susceptibility to neurological diseases (Table 1).

## 3. Parkinson’s Disease

That oxidative stress plays a role in Parkinson’s disease (PD) pathogenesis has been firmly established. Several factors, both genetic and environmental, likely coalesce to deliver a particularly notable oxidative assault upon the midbrain substantia nigra pars compacta (SNpc), whose resident dopaminergic neurons deteriorate over the course of PD. Chief among these factors is the pro-oxidative metabolism of dopamine (DA). DA, which fails to be sequestered in synaptic vesicles following neurotransmission, is degraded in the cytosol by monoamine oxidase in catalysis that yields hydrogen peroxide and 3,4-dihydroxyphenylacetaldehyde (DOPAL), the latter of which may be further oxidized to hydroxy acid [61]. DA may also be oxidized by other enzymes and non-enzymatically by transition metals such as iron. Interestingly, post-mortem analyses have evidenced increased nigral Fe(III) content, especially among patients with advanced PD [62]. While the chemical intermediates of DA oxidation are thoroughly described elsewhere [61], it should be noted that DA quinones have been implicated in mitochondrial dysfunction by forming adducts with proteins such as the γ subunit of ATP synthase [63] and the E3 ubiquitin ligase Parkin [64], inducing mitochondrial permeability transition [63,65], and inhibiting complex I [66]. Further, the ultimate product of DA oxidation, neuromelanin, accumulates in nigral cells with healthy aging and even possesses neuroprotective functions by virtue of its ability to prevent oxidative depletion of the antioxidant ascorbate and sequester iron to prevent the formation of DA quinones [67], and it has been shown to trigger apoptosis in cells overexpressing synuclein [68]. Indeed, the genetic context of PD sets the tone for how oxidative stress influences neurodegeneration. Mutations in the genes *PRKN*, *PINK1*, and *DJ-1*, all inherited in an autosomal recessive manner and directly linked with mitochondrial dysfunction, as well as autosomal dominant mutations in the genes *LRRK2* and *SNCA,* have been implicated in the ‘oxidative stress’ hypothesis of PD etiology [69]. Nonetheless, in a series of elegant experiments using human-induced pluripotent stem-cell-derived DAergic neurons homozygous for a DJ-1 loss-of-function mutation, which in mouse models does not result in decreased nigral DA or α-synuclein aggregation and CRISPR–Cas9-mediated DJ-1 KO cells, the Krainc research team demonstrated that DA oxidation was sufficient to generate typical PD pathology [70]. Thus, DA oxidative metabolism likely represents a common denominator in PD pathology. As such, research into and clinical awareness of antioxidants for the prevention and attenuation of progression of PD is warranted, especially given that levodopa pharmacotherapy is the mainstay of PD treatment. 

Here, we aim not to provide a comprehensive review of the myriad substances previously investigated for utility as antioxidants in PD. For such analyses, the reader might refer to Park and Ellis [71] and Percário et al. [72]. Rather, we compiled measures of the clinical effectiveness of the commonly investigated antioxidants β-carotene/total carotenoids, α-tocopherol (vitamin E), ascorbate (vitamin C), and urate from prospective cohort studies published over the last ten years (Table 2). While the results we provide fall short of a meta-analysis, they allow for relative comparisons of effectiveness between the different antioxidants and point to the probability that response to antioxidant treatment depends on factors such as sex and ethnic background. Moreover, we attempt to draw connections between the mechanisms of action of the antioxidants investigated, focusing particularly on the role of the transcription factor Nrf2. Nrf2 overexpression in astrocytes has been shown to protect against the PD-inducing toxins 6-OHDA [73] and MPTP [74] and promote the lysosomal degradation of mutant α-synuclein aggregates in vivo [75]. We also dedicate particular attention to the Nrf2-modulating antioxidant urate insofar as significant clinical benefits following its use were reported by all of the studies we explored (Table 2).

### 3.1. Urate

Produced by xanthine oxidase in the final step of purine catabolism, urate constitutes the major form in which the body eliminates nitrogenous waste. Early hypotheses into the physiological role of urate noted that the gene responsible for urate degradation, urate oxidase, had been lost during primate evolution. This occurrence was ultimately tied to the phenomenon of increasing primate lifespan thought to be afforded by mechanisms countering prosenescent oxidative changes [76]. Foundational research on urate evidenced that it protected erythrocytes from the ROS generated by heme oxygenase and was neuroprotective with respect to cortical and striatal neurons subjected to ischemia following middle cerebral artery occlusion in rats [77,78]. Nonetheless, it was over a decade after the discovery that heme oxygenase 1, similar to other phase II proteins, is induced via the Nrf2-mediated transactivation of antioxidant response elements (AREs) [78,79] that researchers elucidated that the neuroprotective effects of urate stem largely from its ability to increase astrocytic GSH synthesis [80] in an Akt/GSKβ-dependent manner [81]. In addition, urate has been shown to increase the ratio of the mitochondrial anti/pro-apoptotic proteins Bcl-2/Bax in cells expressing mutant superoxide dismutase and decrease levels of the mutagenic deoxynucleotide 8-OHdG in the cortices of rats having undergone focal cerebral ischemia/reperfusion [81,82]. The latter finding is particularly important in the context of PD insofar as mitochondria, often impaired by PD-associated mutations, have a decreased basal ability to resist mutagens compared with nuclei [83]. That urate might be therapeutic in PD was likely surmised upon discovering that it is decreased in the PD brain [84]. Cipriani et al. [85] demonstrated that exogenously applied urate attenuated DAergic neuron loss in cultures derived from mouse ventral mesencephalic cells treated with MPP+, while reduced levels of endogenous urate, generated via urate oxidase overexpression, made DAergic neurons more susceptible to the toxin. Importantly, astrocyte-enriched cultures in the study cultivated a greater response to urate treatment, corroborating the results of studies examining Nrf2 overexpression in astrocytes mentioned earlier. In addition to its effect on astrocytes, urate has also been found to inhibit microglial pro-inflammatory changes in an experimental model of PD [86]. In the clinical sphere, increased plasma levels of urate were significantly associated with non-penetrance of PD in carriers of the LRRK2 mutation [87]. Importantly, increasing urate for therapeutic benefit in PD was recently deemed safe and shown to be possible via the administration of inosine [88].

### 3.2. Vitamin E

The antioxidant potential of vitamin E for preventing PD development and/or progression has long been recognized. Indeed, over three decades ago, the potential for vitamin E, alone or in combination with diprenyl, to increase the duration of time until patients at an early stage of PD required levodopa treatment was studied in the large, double-blind, placebo-controlled DATATOP clinical trial [89]. While the results of that trial suggested that vitamin E alone did not produce any significant clinical benefits [90] or influence CSF amyloid or tau species levels [91], a recent meta-analysis that included the cohort studies we examine here yielded a pooled odds ratio (OR) of 0.799 for PD development when low versus high vitamin E intake groups were compared [92]. It must be noted, however, that OR < 1 was reported only for Swedish cohorts (Table 2) [93,94]. Moreover, Yang et al. [93] found that a significant OR existed only for women. Similarly to urate, vitamin E was shown to promote levels of the antiapoptotic factor Bcl-xL in a neuronal cell line exposed to hydrogen peroxide and increase Nrf2 expression in those same cells, albeit only under control conditions and not following oxidative stress [95]. In addition, the ability of vitamin E, but not vitamins A, B9, C, or carotenoids, to restore corticostriatal plasticity in a PINK1−/− KO model of PD was recently demonstrated [96].

### 3.3. Vitamin C

As with vitamin E, significant ORs for the development of PD were reported only for Swedish cohorts (Table 2) [93,94]. Moreover, Yang et al. [93] only identified clinical benefits among women and at borderline significance. Somewhat accordingly, the meta-analysis suggesting that vitamin E supplementation may carry therapeutic potential referenced earlier failed to identify any significant benefit associated with vitamin C use [92]. It should be noted that, unlike GSH, ascorbate levels have not been reported as diminished in basal ganglia affected by PD [97]. The therapeutic utility of vitamin C in the context of PD has largely been investigated in relation to the antioxidant’s effect on nigral iron chemistry. While it might be expected to prevent iron-mediated DA oxidation in theory, a recent kinetic analysis evidenced that the higher affinity of DA toward iron and the higher relative stability of Fe(III)DA2 complexes make vitamin C unlikely to reduce DA-bound iron [98]. This accords with a post-mortem analysis, which reported that nigral iron occurred predominantly in the Fe(III) form [97]. Moreover, ascorbate-mediated reduction of Fe(III) to Fe(II), which can generate hydroxyl radicals through the Fenton reaction, may increase oxidative stress, especially given the higher nigral iron content among those with PD. Coupling vitamin C with an iron chelator might have clinical utility based on these results. Interestingly, urate is known to chelate iron. Nonetheless, serum uric acid levels were not found to be associated with nigral iron content [99].

### 3.4. Carotenoids

Significantly decreased odds of PD development with carotenoid use were only reported for women in the Swedish cohort among the observational studies we examined [93]. These results seem to accord with those of a recent meta-analysis that failed to identify a significant pooled OR for PD development with carotenoid use [100]. Importantly, however, β-carotene was shown to be safer compared to its derivative, vitamin A, the latter of which may function pro-oxidatively [101], and should be preferentially selected in the case that carotenoid supplementation is recommended. β-carotene [102], such as the xanthophyll carotenoids fucoxanthin [103] and lutein [104], exerts neuroprotective effects via Nrf2-mediated pathways. Lutein, similar to both urate and vitamin E, has also been shown to protect DAergic neurons from MPTP-induced apoptosis by increasing the ratio of Bcl-2/Bax [105].

**Table 2:** Association between carotenoids, vitamin E, vitamin C, and urate and risk of Parkinson’s disease development based on observational study data.

Antioxidant	Stratification	Confounding Variables Adjusted	Clinical Outcome	Statistical Measure of Outcome *	Value **	*p*-Value **	Reference
ß-carotene	Sex (Women)	Multivariable adjustment	PD development	HR	0.86	<0.01	Yang et al., 2017 [93]
	Sex (Men)	Multivariable adjustment	PD development	HR	0.91	0.05	Yang et al., 2017 [93]
		Multivariable adjustment	PD development	HR	1.05	0.85	Hantikainen et al., 2021 [94]
Total carotenoids		Multivariable adjustment	PD development	HR	0.98	0.83	Ying et al., 2020 [106]
		Multivariable adjustment	PD development	RR	0.97	0.82	Hughes et al., 2016 [107]
Vitamin E	Sex (Women)	Multivariable adjustment	PD development	HR	0.87	0.02	Yang et al., 2017 [93]
	Sex (Men)	Multivariable adjustment	PD development	HR	0.93	0.23	Yang et al., 2017 [93]
		Multivariable adjustment	PD development	HR	0.68	<0.01	Hantikainen et al., 2021 [94]
		Multivariable adjustment	PD development	HR	1.23	0.38	Ying et al., 2020 [106]
		Multivariable adjustment	PD development	RR	0.98	0.82	Hughes et al., 2020 [107]
Vitamin C	Sex (Women)	Multivariable adjustment	PD development	HR	0.91	0.04	Yang et al., 2017 [93]
	Sex (Men)	Multivariable adjustment	PD development	HR	1.02	0.23	Yang et al., 2017 [93]
		Multivariable adjustment	PD development	HR	0.68	<0.01	Hantikainen et al., 2021 [94]
		Multivariable adjustment	PD development	HR	1.3	0.1	Ying et al., 2020 [106]
		Multivariable adjustment	PD development	RR	0.9	0.93	Hughes et al., 2016 [107]
Total antioxidant capacity	Sex (Women)	Multivariable adjustment	PD development	HR	0.93	0.35	Yang et al., 2017 [93]
	Sex (Men)	Multivariable adjustment	PD development	HR	1	0.97	Yang et al., 2017 [93]
		Multivariable adjustment	PD development	HR	0.79	0.16	Hantikainen et al., 2021 [94]
Urate		Multivariable adjustment	PD development	HR	0.56	<0.01	Kobylecki et al., 2018 [108]
		Age and sex	PD development	OR	0.48	<0.01	Bakshi et al., 2019 [87]
		Multivariable adjustment	PD mild cognitive impairment (MCI)	OR	0.55	<0.01	Huang et al., 2018 *** [109]
		Multivariable adjustment	Fatigue in PD	OR	0.69	0.04	Huang et al., 2018 *** [109]

* Statistical measures of outcome are provided for extreme tertiles, quartiles, or quintiles of baseline plasma concentrations of given antioxidants in the studied cohort. Hazard ratio, HR; odds ratio, OR; relative risk, RR. ** Statistically significant results are bolded. *** Only statistically significant non-motor clinical outcomes from this publication are provided. OR for PD-MCI impairment was statistically significant only after MDS level I (abbreviated) but not level II (comprehensive) neuropsychological evaluation.

## 4. Alzheimer’s Disease

As the most common form of dementia, Alzheimer’s disease (AD) represents a significant global health problem that has remained stubbornly resistant to the numerous therapeutic interventions developed to combat it [110]. Indeed, over thirty phase 3 clinical trials have failed to generate effective treatments to reverse or mitigate AD pathology and symptoms [111]. On a biological level, AD is characterized by the extracellular aggregation of amyloid β (Aβ) and intracellular aggregation of hyperphosphorylated tau (pτ), both of which impair cellular function. While research has fixated on these pathological changes as targets for therapeutic intervention, increasing evidence suggests that Aβ and pτ are the products of a complex pathogenesis that must be halted considerably earlier to avoid progressing to a largely irreversible state of neurodegeneration.

Myriad theories have been forwarded to explain AD pathogenesis, ranging from those implicating an infectious etiology [112,113] to others suggesting that it is ultimately a metabolic disorder deserving of the name ‘type 3 diabetes’ [114,115]. Here, however, we focus on the already highly substantiated oxidation–inflammation thesis, which postulates that AD pathology predominantly arises from an imbalance between antioxidant activity and oxidative stress that derives from and exacerbates inflammatory responses [116,117]. Several lines of evidence point to oxidation and inflammation as the culprits in the slow cascade of events leading toward AD neurodegeneration.

**Figure 2:**
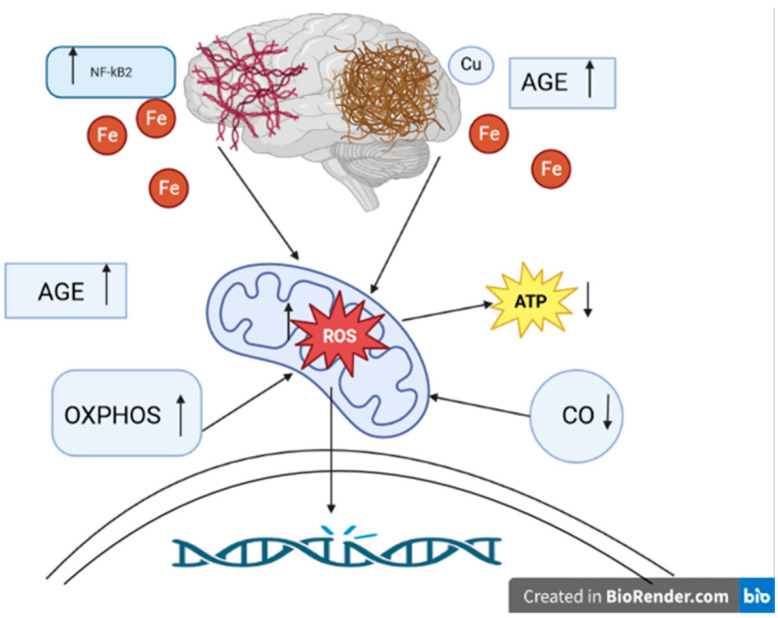
The role of oxidative stress in the pathogenesis of Alzheimer’s disease. ROS: reactive oxygen species. ATP: adenosine triphosphate. NFκB: nuclear factor-kappa B. OXPHOS: nuclear-encoded oxidative phosphorylation subunits. AGE: advanced glycation products. CO: cytochrome c oxidase. Fe: iron ions. Cu: copper ion. ↑ level/activity increase, ↓ decrease in level/activity. Aβ plaques and hyperphosphorylated tau protein tangles play an essential role in the pathogenesis of Alzheimer’s disease. They are most likely involved in generating ROS. Overproduction of ROS can cause permanent DNA damage. The main source of ROS is the mitochondria. ROS can lead to mitochondrial dysfunction, such as decreased ATP levels. Another effect of oxidative disorder is the accumulation of AGE. AGE were noted to be present in Aβ plaques. NFκB activation may be involved in hyperphosphorylation of the tau protein. In Alzheimer’s disease, OXPHOS genes are overexpressed, resulting in increased production of ROS. CO is one of the key elements of mitochondrial metabolism. In Alzheimer’s disease, reduced CO activity is observed with an increase in ROS levels. It is also indicated to shorten the metabolism of metals, e.g., Fe, Cu ions. Disturbances in metabolism can lead to increased production of ROS. The presence of Fe was demonstrated near Aβ plaques and tau protein tangles. Cu, just as Fe, can colocalize with Aβ.

We have previously reviewed how mitochondrial dysfunction, both at the organellar level and that of individual respiratory complexes, has been strongly associated with AD [83]. Increased activity of SOD1 has been observed in familial AD cell lines [118], and studies have demonstrated that crossing mice with a deleted copy of the SOD2 gene with those engineered to be models of AD changed the tissue distribution of Aβ plaques in the CNS and ultimately accelerated AD pathology [119,120]. Further, overexpression of SOD2 was found to prevent AD-associated cognitive decline by lowering hippocampal superoxide levels [121]. In addition, the discovery that iron levels are increased in AD brains and that iron co-localizes with Aβ and pτ, which was confirmed via both imaging and histological analyses, led to the postulation that iron Fenton chemistry is a major source of the ROS that causes macromolecular damage in AD (Figure 2) [111]. Ferroptosis, a programmed cell-death pathway that begins with iron-dependent plasma membrane phospholipid peroxidation, has also been linked to AD. As such, iron chelation represents a tangible target in AD treatment development. Moreover, reducing phospholipid peroxidation by increasing the activities of Se-dependent GPX4 or the newly characterized CoQ-dependent ferroptosis suppressor protein 1 (FSP1) could prove therapeutic [122]. Copper has also been identified as a major player governing the balance between ROS production and elimination in AD. Amyloid precursor protein (APP) knock-out mice were found to have higher levels of copper in the brain, while mice engineered to overexpress APP had decreased levels [123]. Interestingly, while higher copper levels were found to decrease the conversion of APP to Aβ under physiological conditions, oxidation of APP–Cu(I) complexes by hydrogen peroxide was shown to lead to APP fragmentation and subsequent polymerization of Aβ monomers. The latter processes were shown to be reversed by the administration of the copper chelator clioquinol. Seminal research from decades ago established that Cys deprivation lowered GSH levels and promoted cell death, which was amenable to rescue with α-tocopherol (vitamin E) [124].

**Figure 3:**
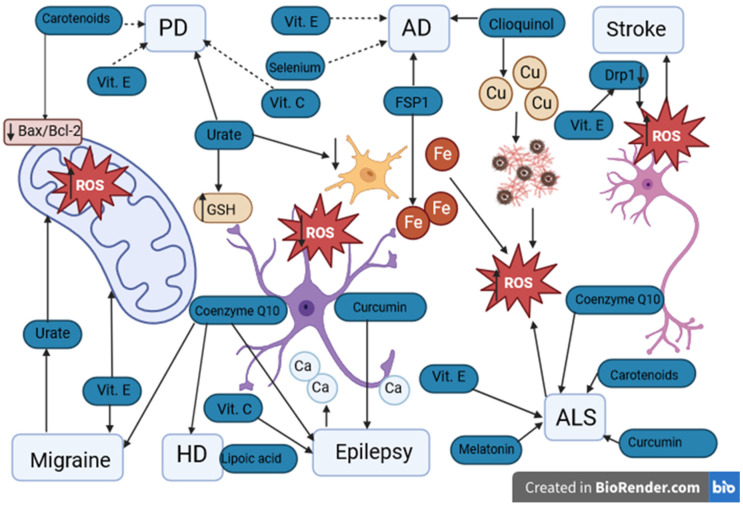
Potential antioxidants used in neurological diseases. PD: Parkinson’s disease. AD: Alzheimer’s disease. HD: Huntington’s disease. ALS: amyotrophic lateral sclerosis. Vit. E: vitamin E. Vit. C: vitamin C. ROS: reactive oxygen species. GSH: glutathione. FSP1: ferroptosis suppressor protein 1. Drp1: dynamin-related protein 1. Fe: iron ions. Cu: copper ions. Ca: calcium ions. ↑ level/activity increase, ↓ decrease in level. Urate has a protective effect on PD by increasing the concentration of GSH in astrocytes and reducing oxidative stress and inflammation in microglia. The protective effect of vitamins E and C in PD remains debatable. In AD, the protective effect of FSP1 occurs by the chelation of Fe ions and clioquinol by the chelation of Cu. The protective effect of vitamin E and selenium in AD has not been confirmed. In ischemic stroke, elevated levels of Drp1 may lead to the death of nerve cells. Decreased levels of Drp1 are noticeable with antioxidants such as vitamin E. Both in migraine and epilepsy, ALS and HD, the protective effect of antioxidants such as vitamins C and E, coenzyme Q10, melatonin, and curcumin is indicated. However, their protective effect requires further research.

Since then, whether supplementation with antioxidants has any bearing on preventing or attenuating AD has received considerable attention. While multiple prospective epidemiological studies have yielded equivocal results [125,126,127,128], a double-blind, multicenter randomized controlled trial showed that supplementation with 2000 IU of vitamin E daily led to significantly increased event-free survival of patients with moderate AD over two years, as compared with placebo (Figure 3) [129]. Unfortunately, this same supplement regimen was not shown to decrease the rate of progression from mild cognitive impairment (MCI) to AD in another double-blind study [130]. More recently, the PREADViSE RCT-turned-cohort study evidenced that neither vitamin E nor selenium prevented dementia in initially asymptomatic men who were followed for a total of 13 years.

## 5. Ischemic Stroke

Stroke is one of the most important factors contributing to long-term disability and death [131]. In the United States there are 795,000 American stroke patients every year [132]. Stroke may be defined as a state of impaired cerebral perfusion. We distinguish stroke caused by an obstruction in arterial and by haemorrhagic incident in the brain [133]. The majority of stroke cases are caused by thrombotic or embolic events [134,135]. During the ischemic stroke, we can distinguish two main brain territories: The ischemic core—where the damage is the most severe and the penumbral area [136]. In general, the interim block of blood flow leads to a lot of metabolic changes, the most notable being Ca^2+^ overload, growth toxicity of glutamate, and apoptosis or necrosis. Neurons of the penumbral area may maintain metabolic homeostasis and survive or enter into apoptosis through the proapoptotic pathways [137]. Thus, this particular area of the brain is the aim of therapeutic strategies in the post-stroke period and crucial steps should be taken to minimize the total volume of infarct.

Mitochondria are small intracellular organelles with a double membrane. The inner membrane is the center of the metabolism and contains complexes used for electron transport, ATP/ADP proteins, oxygen, carbon dioxide, and water. The major role of mitochondria is to generate cellular energy in the form of ATP. That purpose is fulfilled by the mitochondrial electron transport chain through oxidative phosphorylation [138]. Under normal circumstances, the higher part of cerebral ATP is involved in maintaining neuronal electrogenic activity [139]. Additionally, mitochondria are the main source of ROS in mammalian cells that can contribute to the regulation of apoptotic processes [140,141]. ROS are formed in response to the reperfusion phase by mitochondrial complex I, xanthine oxidase, cyclooxygenases/lipoxygenases-, and phagocyte NADPH oxidases [142,143,144,145]. Excess production of ROS relative to antioxidant defense leads to the generation of oxidative stress. The level of ROS is increased after an episode of cerebral ischemia, which was illustrated with hydroethidine [146]. This situation is caused by the unsaturation of cytochrome c oxidase at the terminus of the electron transport chain and the hyperactivity of complex I and III, which gives the increased level of ROS [147]. It is shown that ROS may interact with NF-κB, which gives opposing effects on NF-κB activation in the cytosol and the nucleus [148,149]. Furthermore, the ROS-induced activation of NF-κB may result in the synthesis of target messenger RNASs (mRNAs), which is a stimuli to proinflammatory cytokines production [150]. Additionally, resident microglia produce tumor necrosis factor alpha (TNF-α), interleukin-1beta (IL-1β), and interleukin-6 (IL-6) [151]. Supposedly, microglia activation contributes to neuronal death by mitochondrial dysfunction [152,153]. TNF-α causes suppression of mitochondrial complexes I, IV, and pyruvate dehydrogenase [153,154,155,156]. IL–6 increases the production of ROS [156] and nitric oxide inhibits complex IV [157,158]. It is demonstrated that NF-B is also produced by microglia [159,160]. All of these processes may explain the role of mitochondria during reperfusion after cerebral ischemia, additionally confirming the important role of mitochondria during the ROS cascade and inflammation-induced neurotoxicity [161].

The latest studies show that after hours or days, neurons in the penumbra area may undergo apoptosis [162,163,164]. Stopping that process gives a chance to limit infarct volume after a stroke. After the cerebral ischemia, the increased level of ROS may trigger molecular pathways. Disruption of mitochondria and release of the proapoptotic proteins may shift cell biology into apoptosis pathway [165]. The main role in apoptosis comes from a Bcl-2 protein family, which is a major regulator of outer mitochondrial membrane permeability [166]. After cerebral ischemia, the level of proapoptotic BH3-only Bcl-2 subfamily is increased, which may confirm the stroke-induced activation of multiple apoptotic pathways binned with mitochondria [167,168]. Just before apoptosis, mitochondria break apart into small units. It is believed mitochondrial fission precedes neuron death after cerebral ischemia [169]. The dynamin-related protein 1 (Drp1) is a factor leading to an imbalance of mitochondrial fission and fusion. After mitochondrial oxidative stress, the level of Drp1 is increased, which may lead to cell death [170]. A reduced level of Drp1 is noticeable with antioxidants, e.g., vitamin E and MitoQ (Figure 3) [171,172]. Furthermore, the low level of Drp1 decreases ROS production and mitochondrial stress [173]. In consideration of this, the downregulation of Drp1 may reduce the infarct volume, which was confirmed in a focal cerebral ischemia model [174,175]. Cardiolipin, diacylglycerol, lysophosphatidic acid, phosphatidylethanolamine, and phosphatidic acid may have a role in mitochondrial fission and fusion, but their potential beneficial effect needs more studies [176].

Apart from vitamin E and MitoQ mentioned above, there are several endogenous and exogenous antioxidants that may play a crucial role in stroke [177]. The study of Arts and Hollman [178] proved that increased consumption of polyphenols (quercetine, kaempferol, myricetine, apigenine, and luteolin) may lower potential stroke risk. Another exogenous substance that may serve as a protective agent in stroke is selenium [179]. This mineral stimulates selenome via the activation of transcription factor AP-2 gamma (TFAP2c) and special protein 1 (sp1). As a result, the GPX4 activity is greatly increased, which inhibits ferroptosis significantly and lowers stroke-induced neuronal damage [179,180]. Uric acid, on the other hand, is one of the endogenous agent involved in stroke. Elevated level of uric acid is correlated with ischemic stroke [181,182]. It is proposed that severe hypertension might be the intermediate path between hyperuricemia and stroke. This may be further explained by epidemiological, animal, and clinical trials [183,184,185,186,187].

Another pathology potentially responsible for cell death after stroke is ferroptosis. Recent studies have shown increased association between pathological ferroptosis activity and intracranial aneurysm along with hemorrhagic stroke [188,189]. There are three crucial systems preventing excessive ferroptosis, the GPX4–GSH–cysteine system, GCH1–BH4–DHFR system, and FSP1–CoQ10–NAD(P)H system. The imbalance in those systems induced by stroke may lead to further neuronal damage [190]. Disruption in glutamate intake, cystine/glutamate antiporter xC inhibition, and, as a result, downregulated production of glutathione (GSH) may lead to inactivation of GPX4, ultimately promoting ferroptosis [191,192]. Additionally, the appearance of reactive-species-generating compound 3 (RSL3), which is a direct inhibitor of GPX4, may further hinder the cellular antioxidant abilities, resulting in ROS accumulation and excessive ferroptosis [191,193]. As a matter of fact, a new thesis has emerged that ferroptosis inhibitors might have therapeutic potential in neurodegenerative diseases, brain injuries, and stroke [194,195].

## 6. Other Neurological Diseases

Neurological diseases in which there is an imbalance between the concentration of ROS and the natural ability to detoxify reactive products of metabolism also include diseases such as migraine, epilepsy, amyotrophic lateral sclerosis (ALS), and Huntington’s disease (HD).

Migraine is one of the most common neurological disorders [196]. The estimated prevalence of migraine is about 12% of the population, and is higher in women than men (3:1) [197]. According to the 2018 ICHD-3 classification, migraine can be divided into two clinical forms: migraine with aura (MA) and migraine without aura (MO) [198].

Despite the high frequency of migraine, its causes are still not fully understood. Migraine is considered a multifactorial and polygenic disease. In the pathogenesis of migraine, particular attention is paid to the biochemical theory, cortical-spreading depression (CSD), and the theory of neurogenic inflammation in the TGVS system. The biochemical theory assumes that during headache attacks, there are fluctuations in the concentration of serotonin in plasma, which leads to cerebral vasoconstriction [199]. A second theory of migraine pathogenesis involves CSD. CSD is associated with MA [200]. According to this theory, pain is caused by a slow wave of depolarization of neurons and glial cells, followed by their prolonged suppression [201]. The third described pain mechanism in migraine is neurogenic inflammation in TGVS.

The role of oxidative stress in migraine is still unclear. The association of oxidative stress and mitochondrial dysfunction with the TGVS theory seems most likely due to the neurogenic inflammation in this mechanism. According to a 2013 study by Shatillo et al. [202], ROS can directly stimulate trigeminal nociceptors (TG) and increase the release of calcitonin gene-related peptide (CGRP) from TG’s C fibers. Due to the lack of the blood–brain barrier (BBB), CGRP is released into the circulation, where it causes the dilation of cerebral and dural blood vessels [203]. As a consequence, there is a transient increase in cortical blood flow, as well as stimulation of glial cells to release proinflammatory factors, such as TNF-α, IL-1β, and prostaglandins E2 (PGE2), ultimately causing neuroinflammation [204,205]. The release of these compounds is also stimulated by nitric oxide (NO). NO is released from TG neurons into the intercellular space along with CGRP. NO molecules, just as CGRP, stimulate glial cells to release TNF-α, IL-1β, and PGE2 [204]. At the same time, NO is known as a biomarker of oxidative stress [206]. A study by Bulboacă et al. [207] from 2020 confirmed elevated values of the oxidative stress parameters NO and malondialdehyde (MDA) in people with migraine compared to controls. The authors indicate excess NO reacts with superoxide to form peroxynitrite (ONOO−). Peroxynitrite is a harmful by-product of mitochondrial respiration [208]. Peroxynitrite can irreversibly bind transition metals necessary for enzymatic and cell reactions. For example, if peroxynitrite blocks iron located in the iron–sulfur center of one of the enzymes of the respiratory chain (e.g., complex I), energy production in the mitochondria decreases, and their general dysfunction of neuron cells occurs [209].

Oxidative stress can also be combined with cortical-spreading depression (CSD). Jiang et al. [210] conducted a study to analyze the relationship between ROS and CSD in migraine. This study was conducted with an animal model. The authors concluded that ROS plays a crucial role in evoking CSD by regulating the activity of the TRPA1 ion channel. In addition, they confirmed that the inhibition of ROS activity by an antioxidant reduces the probability of CSD in vivo [210,211].

Due to the possible influence of ROS on both mechanisms of migraine formation, the importance of antioxidants for migraine patients seems to be important. Yang et al. [212] conducted a study for 148 migraine patients that showed differences in concentrations of several major intravascular antioxidants, such as serum albumin (ALB) and creatinine (CRE), bilirubin (BIL), and uric acid (UA). The serum levels of ALB, total BIL (TBIL), CRE, and UA were significantly lower in the migraine group than in the control group. Moreover, the ALB and UA levels were lower during migraine attack periods (*p* < 0.05). For example, UA is an endogenous antioxidant that can reduce oxidative damage and inhibit inflammatory responses, reducing BBB permeability and having neuroprotective effects [213]. Moreover, UA is a specific inhibitor of radicals generated by the decomposition of peroxynitrite (ONOO), so it protects the mitochondrial respiration chain [214]. The authors hypothesized that the lower levels of these essential antioxidants might be due to their depletion by neutralizing the ROS molecules that circulate in increased concentration throughout the migraine sufferer’s body [212].

One of the most important exogenous antioxidants for migraine is curcumin. Curcumin is a natural polyphenol compound found in turmeric [215]. Bulboacă et al. [216] showed that curcumin exhibits anti-inflammatory properties by inhibiting cyclooxygenase (COX-s) and inhibiting the expression of interleukin: IL-1 and IL-6. These relations are significant in the context of the theory of neurogenic inflammation involving TGVS as the genesis of migraine headaches. In the final stages of TG inflammation, neurons release CGRP and NO into the extracellular spaces. NO further stimulates astrocytes to produce COX and interleukins 1 and 6 as the primary triggers of inflammation in the body [214]. Curcumin is, thus, an inhibitor of NO function. By inhibiting the activity of COX and the expression of Il-1 and Il-6, curcumin could significantly reduce the formation of pain in migraines.

A well-known antioxidant effective in migraine is vitamin E. Vitamin E inhibits the release of arachidonic acid and the conversion of arachidonic acid from cell membranes to prostaglandin (PG) by acting on the enzymes phospholipase A2 and cyclooxygenase (COX) [217]. Thus, it limits the participation of PG in TGVS neurogenic inflammation.

In addition, coenzyme Q10 is also mentioned in the prevention of migraine as one of the most important endogenous antioxidants. It protects the mitochondria against the action of free radicals because it directly transfers electrons between complex I and complex II of the respiratory chain [218]. In addition, CoQ10 is the only known fat-soluble antioxidant that can be synthesized de novo by animal cells and for which there are enzymatic mechanisms that can regenerate the antioxidant from its oxidized form, resulting from its lipid peroxidation-inhibiting effect [218,219].

Epilepsy is a neurological disease with a permanent predisposition to generate epileptic seizures [220]. Seizures are time-limited paroxysmal events that result from abnormal, involuntary, rhythmic neuronal discharges in the brain. The crucial role in the disease development is the formation of the epileptogenic focus, which has uncontrolled hyperexcitability due to prolonged partial depolarization of cellular membranes. Seizures can result in neuronal cell death through dynamic processes that might include genetic factors, excitotoxicity-induced mitochondrial dysfunction, altered cytokine levels, oxidative stress, and changes in plasticity or activation of some late cell death pathways. The formation of reactive species or the decreased activity of antioxidant systems may result in different forms of epilepsy and increased chances of repeating epileptic seizures [221,222].

Oxidative stress, which may contribute to seizures, may result from abnormal levels of Ca^2+^ ions in astrocytes. Ca^2+^ ions are released from the endoplasmic reticulum when astrocytes are stimulated [223]. Ding et al. found increased Ca^2+^ activity in astrocytes in the days following pilocarpine-induced epileptic seizure in mice [224]. In addition, a study by Pandit et al. [225] from 2020 showed that stimulated astrocytes, in which the concentration of Ca^2+^ ions was increased, were characterized by increased release of the neurotransmitter GABA through the bestrophin-1 channels. These channels are activated by Ca^2+^ ions. Kovacs et al. [226] showed that the accumulation of mitochondrial Ca^2+^ can stimulate the production of NADH and ROS. Overproduction of ROS during an epileptic seizure leads to mtDNA damage and reduces the activity of mtDNA-encoded mitochondrial electron transport chain (ETC) subunits, notably CI, CIII, CIV, and CV, which are involved in the cellular respiration chain. Reduced activity of ETC subunits may lead to the circulation of free electrons in the mitochondria, forming free radicals and causing oxidative stress.

In the case of epilepsy, the most important antioxidant seems to be coenzyme Q10, also called ubiquinone. Coenzyme Q10 is a powerful antioxidant that reacts with ROS and the accumulation of Ca^2+^ ions in the mitochondria. ROS neutralization prevents oxidative damage to cells caused by free radicals, including lipid peroxidation of the mitochondrial membrane. According to Santos et al. [227], in a rat model of epilepsy, the administration of ubiquinone to animals increased the activity of the antioxidant enzymes SOD, CAT, and GPx. It can be concluded that ubiquinone not only acts as an antioxidant on its own but also supports the action of other antioxidant compounds. In addition, antioxidants such as vitamin C and curcumin are essential in reducing oxidative stress in epilepsy. Curcumin, as in migraine, removes ROS and RNS, which may result from the accumulation of Ca^2+^ [228,229,230]. Vitamin C, acting similarly to CoQ10, increased the activity of the antioxidant enzymes SOD and CAT [222].

ALS is the most common motor neuron disease [231]. It is an incurable, progressive disease that is caused by a selective loss of upper motor neurons (UMN) of the motor cortex and lower motor neurons (LMN) of the brainstem and spinal cord [232]. The clinical picture of an ALS patient includes weakness, numbness, and atrophy of the limb or bulbar muscles, ultimately leading to paralysis of the respiratory muscles and respiratory failure, leading to death [233]. ALS is divided into two types: familial ALS (fALS) and sporadic ALS (sALS). The causes of sALS are still unknown, but fALS is caused by autosomal, usually dominant mutations [234]. In the pathogenesis of ALS, mutations in the genes encoding the SOD1 and TDP-43 proteins are the most important. Among the mutations in the *SOD1* gene, the A4V missense mutation in exon 16, on chromosome 21q22.11, is important for the pathogenesis of ALS [59]. In addition to SOD1, the enzymatic antioxidant capacity also depends on the activity of GPx and GR enzymes related to GSH. In 2014, Weiduschat et al. [235] confirmed that GSH levels are 31% lower in patients with ALS than in healthy controls. Observed by Weiduschat et al. [235], results indicate that the lower level of GSH in ALS patients significantly weakens their oxidative protection and, at the same time, may be associated with a significant impairment of GSH reproduction by the GR.

The second mutation associated with ALS is a mutation in the *TARDBP* gene, which is present on chromosome 1. The expression product of this gene is the TDP-43 protein, which is present in all cell types, mainly in the cell nucleus but also in the cytoplasm and mitochondria. TDP-43 binds DNA and RNA in the nucleus, thereby regulating transcription, RNA alternative splicing, and miRNA processing [236]. In ALS, a mutation in the *TARDBP* gene causes abnormal phosphorylation and accumulation of TDP-43 in the cytoplasm of motor neurons. Accumulation leads to the formation of cytoplasmic inclusion bodies that deplete nuclear proteins and impair the function of the entire cell [237]. Iguchi et al. [238] emphasized that this accumulation may result from the mutation and GSH deficiency, which causes oxidative stress.

Based on many oxidative stress mechanisms described in ALS, the use of antioxidants, therefore, seems important in the context of delaying progressive muscle paralysis [239]. In the case of ALS, antioxidants such as carotenoids, epigallocatechin gallate (EGCG), and melatonin are important. Carotenoids are natural pigments responsible for fruits and vegetables orange, red, yellow, or green color. Carotenoids have long been credited with antioxidant and ROS-neutralizing properties [240]. In general, O_2_ deactivation is based on converting excess energy into heat via an excited carotenoid (Crt) [241]. Another antioxidant is epigallocatechin gallate (EGCG). EGCG is a catechin in green tea, credited with antioxidant and anti-neurodegenerative effects. EGCG crosses the BBB and modulates the mitochondrial response to ROS [242]. A 2004 study by Koh et al. [243] proved that EGCG prevents ROS-induced SOD1-mutated motor neuron cell death by altering cell survival and death signals.

An example of an exogenous antioxidant for ALS is melatonin. Melatonin is a pineal gland hormone that primarily regulates the circadian rhythm [244]. It is highly effective in reducing oxidative stress by directly detoxifying ROS and RNS and indirectly by stimulating antioxidant enzymes (GPx) while inhibiting the activity of pro-oxidative factors (NO). Moreover, melatonin reaches a high concentration in the mitochondria, giving it a direct antioxidant effect. In addition, among the antioxidants mentioned in ALS, the same as for migraine and epilepsy can be distinguished: curcumin, coenzyme Q10, and vitamin E [245].

Another neurological disease inherited in an autosomal dominant manner is HD. HD is a neurodegenerative disease that results from a mutation in the *HTT* gene, which encodes huntingtin. Huntingtin is located in the cell division spindle. This protein is involved in forming and differentiating new neurons and forming proper connections between them. Mutation of the *HTT* gene causes multiple CAG repeats that translate into an abnormally long polyglutamine repeat in the mutant huntingtin (mhtt) protein [246].

Additionally, excessive expansion of accumulated CAG repeats may be due to mitochondrial oxidative stress. The resulting ROS can oxidize DNA molecules containing more CAG repeats. DNA oxidation mobilizes the activity of repair enzymes (e.g., BER–OGG1 or NEIL1 enzymes), which seek to remove oxidized bases from DNA [247]. However, eliminating these bases can result in an increased expansion of the CAG repeats in the DNA molecule and, consequently, an increased amount of the abnormal huntingtin protein [246]. Toxic gain of functions for the mutant huntingtin protein may include protein aggregation, transcriptional dysregulation, defective energy metabolism, oxidative stress, excitotoxicity, and neuron inflammation [248].

The abnormal mhtt protein has a neurotoxic effect because it accumulates in nerve cells, interfering with mitosis and proper differentiation. The neurotoxicity of the protein is also due to its effect on the mitochondria. Mhtt can cause deletions in mitochondrial DNA (mt-DNA) [249]. Kim et al. [250] highlighted the decreased levels of mitochondrial proteins (Mfn1, Mfn2, Opa1, and Tomm40) found in HD patients. In addition, there were decreased levels of electron transport proteins, cytochrome b, and cytochrome c oxidase 1 in HD patients with the simultaneous presence of mhtt aggregates in their cerebral cortex. Importantly, immunolabelling of brain samples showed the presence of mhtt oligomers in the nuclei of neurons and mitochondria of patients with HD.

Antioxidants in HD include coenzyme Q10, vitamins (C and E), and melatonin, as in ALS. Among the antioxidants important for HD, there are also, for example, selenium and lipoic acid. However, these antioxidants are of supportive importance, as oxidative stress in HD results primarily from impaired mitochondrial function due to mhht protein aggregates [251]. Selenium is an essential element of GPX required to form the active enzyme. Lipoic acid is present in mitochondria as the cofactor for alpha-ketoglutarate dehydrogenase, which participates in the Krebs cycle. Additionally, an Andreassen et al. [252] study using the R6/2 and N171-82Q transgenic mouse models of HD suggested that dietary supplementation with lipoic acid resulted in a significant extension of survival. The authors of this study concluded that these findings indicate that alpha-lipoic acid might benefit HD patients.

Table 3 presents a summary of exogenous and endogenous antioxidants that are important in the above-mentioned selected neurological diseases. As can be seen in this table, regardless of the pathomechanism of the disease, antioxidants such as vitamin C, vitamin E, curcumin, melatonin, or coenzyme Q10 seem to be effective in each of these neurodegenerative diseases.

As presented above, oxidative stress is an important factor in neurological diseases. Therefore, antioxidants can play an important role in preventing, mitigating, or delaying the disease’s symptoms (Figure 3). The development of research on this issue may bring us closer to a better understanding of the pathomechanisms of described disorders and to developing a more effective treatment.

## 7. Conclusions

Oxidative stress is caused by an imbalance in the redox system. It can be caused by excessive production of ROS or dysfunction of the antioxidant defense system. Moreover, oxidative stress is one of the causes of the development of most neurological diseases including AD, PD, stroke, ALS, HD, epilepsy, and even migraine. In these diseases, excessive ROS is observed in areas subject to structural damage or other disorders at the cellular level, e.g., neurotransmission. At the same time, impairment of the level/activity of biomarkers of antioxidant defense mechanisms is often observed in these diseases. While research on oxidative stress in the course of neurological diseases is the subject of numerous experimental and clinical studies, research on antioxidants is less numerous. It is not known whether in the case of neurological diseases, the endogenous or exogenous antioxidant level is more disturbed, at what stage of the disease development supplementation should be introduced, and whether these should be forms of drugs, paradrugs, or cofactors and what their dosage is. Moreover, when antioxidants can be most effective and whether they are effective in counteracting ROS is also not fully understood. These issues require further research.

## Figures and Tables

**Table 1:** Genetic variants in antioxidant enzymes conferring increased susceptibility to neurological diseases.

Gene	Gene Name	Chromosomal Location	Genetic Variants	Clinical Effects	References
*CAT*	Catalase	11p13	chr11:34438684C > G/C > T (upstream transcript variant; rs1001179)	Migraine susceptibility	www.ncbi.nlm.nih.gov/clinvar Accessed on 4 July 2023
*GPX*	Glutathione peroxidases	19p13.3	c.660T > A (SNP; rs713041)	Associated with neurodegeneration and susceptibility to stroke	Borchert et al., 2018 [21] www.ncbi.nlm.nih.gov/clinvar Accessed on 4 July 2023
*GSTM1*	Glutathione S-transferase (mu) M1	1p13.3	GSTM1*0 (homozygous deletion)	Associated with AD pathology	Bolt and Their, 2006 [43] Lo et al., 2007 [42] Wang et al., 2016 [41]
*GSTT1*	Glutathione S-transferase (theta) T1	22q11.2	GSTT1*0 (homozygous deletion)	AD risk is present only in Asian populations	Bolt and Their, 2006 [43] Lo et al., 2007 [42] Wang et al., 2016 [41]
*GSTP1*	Glutathione S-transferase (pi) P1	11q13-qte	c.313A > G (SNP; rs1695)	Associated with AD pathology	Bolt and Their, 2006 [43] Lo et al., 2007 [42] Wang et al., 2016 [41]
*SOD1*	Superoxide dismutase-1	21q22.11	A4V missense mutation (NeuroX_21:33032096) 16 exonic mutations	ALS pathogenesis	Andersen et al., 2003 [59] www.varsome.com Accessed on 4 July 2023
*SOD2*	Superoxide dismutase-2	6q25.3	c.47T > C (SNP; rs4880)	Increases PD risk	Singh et al., 2008 [60]
*PRX*	Periaxin	19	2-Cys Prx	Associated with neurodegeneration	www.ncbi.nlm.nih.gov/clinvar Accessed on 4 July 2023
*HMOX1*	Heme oxygenase 1	22	Length polymorphisms in the number of GT dinucleotide repeats in the *HMOX1* promote	Vascular diseases	www.ncbi.nlm.nih.gov/clinvar Accessed on 4 July 2023

AD—Alzheimer’s disease; ALS—amyotrophic lateral sclerosis; PD—Parkinson’s disease.

**Table 3:** Antioxidants in selected neurological diseases. CSD, cortical-spreading depression; ALB, albumin; CRE, creatinine; BIL, bilirubin; UA uric acid; EGGG, epigallocatechin gallate.

Neurological Disease	Damaged Brain Structure	Pathomechanism	Common Antioxidants		Specific Antioxidants		References
			Exogenous	Endogenous	Exogenous	Endogenous	
Migraine	Neurons and glial cells	CSD	Curcumin Vitamin E Vitamin C Melatonin	Coenzyme Q10	-	ALB	van Nueten, 1985 [199] Eftekhari et al., 2015 [203] Yang et al., 2022 [212] Bulboacă et al., 2019 [216] Ziaei et al., 2005 [217] Wang et al., 2016 [218]
	Neurons and glial cells	Neurogenic inflammation				CRE	
	Cerebral and dural blood vessels					BIL	
						UA	
Epilepsy	Neurons	Prolonged depolarization			-	-	Essig et al., 1966 [221] Martinc et al., 2014 [222] Santos et al., 2010 [227] Wang et al., 2010 [230]
Familial amyotrophic lateral sclerosis	Upper motor neurons and lower motor neurons and spinal cord	Mutations in the genes encoding the SOD1 and TDP-43 proteins			Carotenoids	-	Valko et al., 2019 [232] Al-Chalabi et al., 2013 [234] Nisar et al., 2015 [239] Chung et al., 2010 [241] Muñoz et al., 2020 [244]
					EGCG		
Huntington’s disease	Neurons	Mutation in the *HTT* gene			Selenium	Lipoic acid	Jimenez-Sanchez et al., 2017 [245] Johri et al., 2012 [251] Andreassen et al., 2001 [252]
